# Supplementary material for: The causal role of circulating inflammatory markers in osteoporosis: a bidirectional Mendelian randomized study
Source: Front Immunol. 2024 Jul 18;15:1412298. doi: 10.3389/fimmu.2024.1412298 (PMC11291241; doi:10.3389/fimmu.2024.1412298)
Supplement: Supplementary file 1 [file DataSheet_1.zip › supplementary material/OSTEOPOROSIS-CIM/GCST90274842.tsv.gz_forest.pdf]

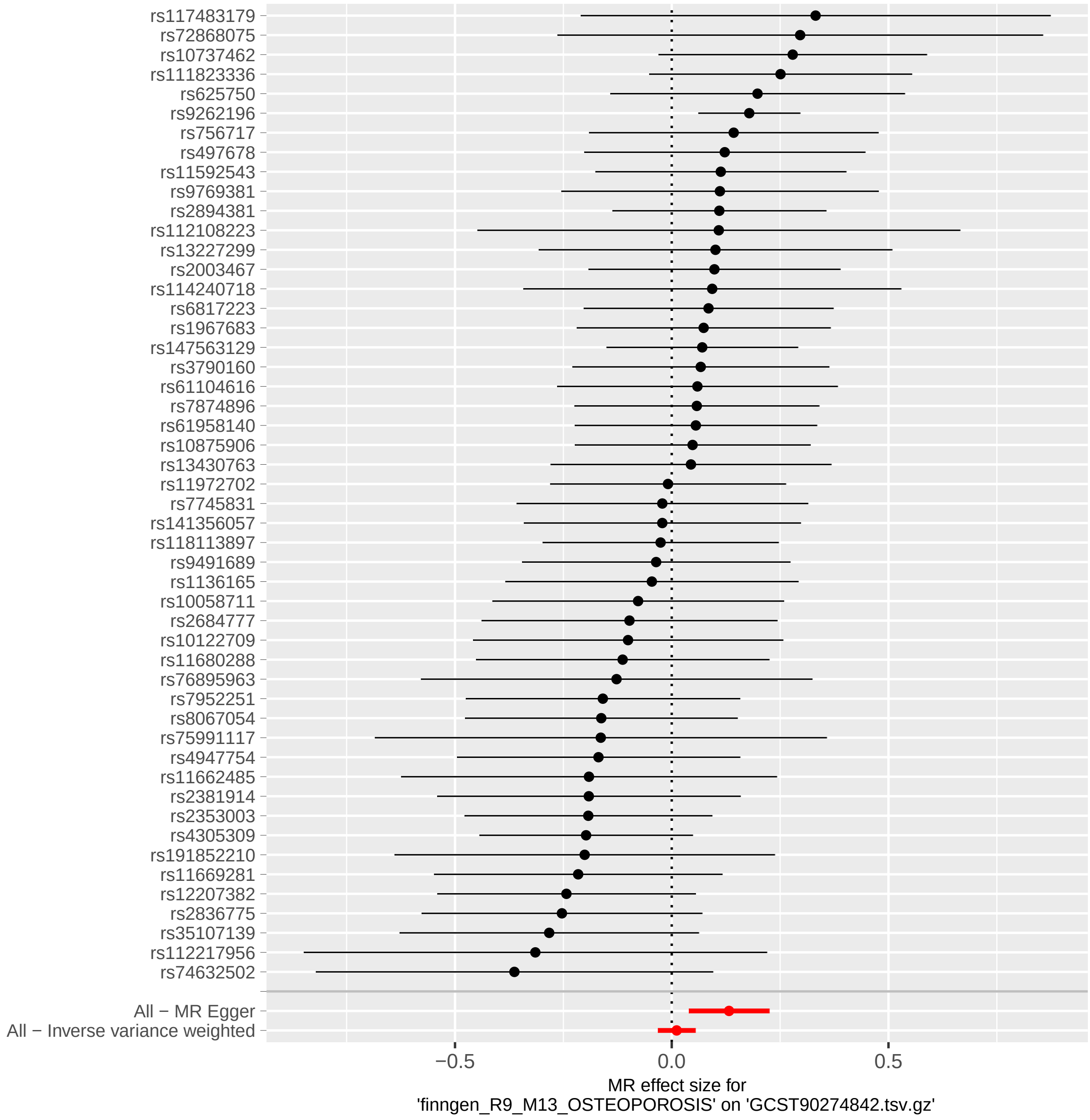
rs117483179
rs72868075
rs10737462
rs111823336
rs625750
rs9262196
rs756717
rs497678
rs11592543
rs9769381
rs2894381
rs112108223
rs13227299
rs2003467
rs114240718
rs6817223
rs1967683
rs147563129
rs3790160
rs61104616
rs7874896
rs61958140
rs10875906
rs13430763
rs11972702
rs7745831
rs141356057
rs118113897
rs9491689
rs1136165
rs10058711
rs2684777
rs10122709
rs11680288
rs76895963
rs7952251
rs8067054
rs75991117
rs4947754
rs11662485
rs2381914
rs2353003
rs4305309
rs191852210
rs11669281
rs12207382
rs2836775
rs35107139
rs112217956
rs74632502
All − MR Egger
All − Inverse variance weighted
−0.5
0.0
0.5
MR effect size for
'finngen_R9_M13_OSTEOPOROSIS' on 'GCST90274842.tsv.gz'
